# Cold Stress-Induced DNA Methylation Profiling and Its Association with Terpenoid Biosynthesis in Germinating *Mikania micrantha* Seeds

**DOI:** 10.3390/epigenomes10040067

**Published:** 2026-10-12

**Authors:** Zijuan Yu, Can Cui

**Affiliations:** 1 School of Landscape Architecture, Guangdong Eco-Engineering Polytechnic, Guangzhou 510520, China; 2 School of Horticulture and Food, Guangdong Eco-Engineering Polytechnic, Guangzhou 510520, China

**Keywords:** *Mikania micrantha*, seed germination, cold stress, DNA methylation, epigenetic regulation

## Abstract

*Mikania micrantha*, a highly invasive weed, faces constraints on its northward range expansion in South China due to low winter temperatures. Although DNA methylation is increasingly recognized as a mediator of plant stress adaptation, it remains unknown whether epigenetic regulation couples cold-stress tolerance with the production of allelopathic secondary metabolites in invasive plants. Here, we comprehensively characterized genome-wide DNA methylation dynamics and their transcriptional consequences in germinating embryos under three temperature regimes (14 °C, 25 °C, and 30 °C) by integrating whole-genome bisulfite sequencing and RNA-Seq. Exposure to 14 °C significantly delayed germination without arresting the process. Global methylation levels were highest at CG sites (80.21–84.63%), followed by CHG (63.67–66.77%) and CHH (6.63–7.51%) contexts. Integrative analysis identified 1103 differentially methylated region-associated genes, of which 434 were also differentially expressed. Notably, KEGG enrichment of the 434 overlapping genes revealed significant overrepresentation of the sesquiterpenoid and triterpenoid biosynthesis pathways, with key mevalonate (MVA) pathway genes showing altered expression in association with DMRs at 14 °C. Because this is an integrative methylome–transcriptome association study without metabolite quantification or functional validation, these results are consistent with the hypothesis that cold-induced epigenetic reprogramming is associated with transcriptional changes in terpenoid biosynthesis, which may contribute to antioxidant and membrane-protective functions under chilling stress; the possible accumulation of allelopathic sesquiterpene lactones and any role in maintaining competitive advantage during cold seasons remain working hypotheses that require experimental validation. The results of this study offer new epigenetic perspectives and supporting data for the management and prevention of *M. micrantha* invasion.

## 1. Introduction

*Mikania micrantha* is renowned for its exceptionally rapid proliferation, often colloquially described as “mile-a-minute” weed, a trait largely attributable to its broad physiological tolerance, vigorous asexual reproduction, allelopathic properties, and the production of copious high-quality seeds [1]. Its seeds are minute and bear pappi, facilitating rapid germination in regions with favorable temperatures [2,3]. As in most seed plants, rapid seed germination and successful seedling establishment are critical during the invasion process of *M. micrantha*. In southern China, flowering and achene production occur from August through May of the following year, whereas the peak period for seedling establishment is typically from September to November, coinciding with optimal germination temperatures of approximately 25–30 °C [4]. Although germination has been reported at 15 °C or 35 °C, temperatures deviating markedly from this optimum significantly suppress both germination and seedling establishment [4,5]. In South China, where *M. micrantha* aggressively invades, winter temperatures frequently drop below 15 °C [6]. In response to cold conditions, this weed often elevates anthocyanin content or modifies its morphological appearance [7]. Combined with current distribution records within China, the species is predominantly found in Guangdong, Guangxi, Hunan, and Fujian provinces, with its northernmost occurrence reaching Fuzhou (Fujian) and showing a tendency for further northward expansion. These lines of evidence suggest that low growth temperatures represent one of the primary constraints limiting its northward spread in China. Nevertheless, studies on the effects of cold stress on seed development in *M. micrantha* remain scarce, particularly with respect to integrated analyses of molecular genetic variation and epigenetic regulation during germination.

Low temperature is a major environmental factor limiting plant growth, productivity, and geographical distribution [8,9]. Plants have evolved sophisticated physiological and molecular adaptations to cold stress, including the CBF/DREB1 transcriptional cascade, accumulation of compatible osmolytes, and modulation of membrane lipid composition [9]. In addition to these well-characterized genetic pathways, epigenetic mechanisms including DNA methylation, histone modifications, chromatin remodeling, and non-coding RNAs play critical roles in regulating stress-responsive gene expression and can provide rapid, reversible, and potentially heritable adaptive responses [10,11,12]. DNA methylation, the most extensively studied epigenetic mark in plants, occurs in three sequence contexts (CG, CHG, and CHH, where H = A, T, or C) and is catalyzed by distinct methyltransferases, including *MET1* (CG), *CMT3* (CHG), and *DRM2* (CHH) via the RNA-directed DNA methylation pathway [13]. Cold-induced changes in DNA methylation have been documented in several plant species: for example, cold priming in tartary buckwheat involves dynamic methylation changes that enhance subsequent cold tolerance [14], and cold acclimation in Brassica rapa alters methylation patterns while conferring cross-tolerance to heat [15]. At the histone level, cold stress triggers rapid redistribution of the antagonistic marks H3K4me3 (active) and H3K27me3 (repressive), and induces bivalent chromatin states that poise stress-responsive genes for rapid activation [16,17]. These epigenetic mechanisms collectively fine-tune the spatiotemporal expression of stress-responsive genes and contribute to stress memory [12].

The role of epigenetics in biological invasions has attracted growing attention. Invasive plant species often establish and spread despite low genetic diversity resulting from founder effects, suggesting that non-genetic sources of phenotypic variation may be particularly important [18,19]. DNA methylation can generate phenotypic plasticity independent of DNA sequence variation, mediate rapid adaptation to novel environments, and especially in clonal species produce heritable epialleles that influence ecologically important traits [18,20]. In *M. micrantha*, population-level studies have revealed significant epigenetic differentiation associated with environmental heterogeneity [21], and our previous work demonstrated that cold-dependent DNA demethylation and antioxidant regulation jointly contribute to restoring seed germination under low temperature [22]. However, the functional targets of cold-induced epigenetic reprogramming in this species, particularly whether methylation changes regulate secondary metabolic pathways relevant to both stress tolerance and invasiveness, remain largely unexplored.

Cold stress induces the overproduction of reactive oxygen species (ROS), leading to oxidative damage to lipids, proteins, and nucleic acids [8]. Beyond enzymatic antioxidant systems, plants accumulate a diverse array of specialized (secondary) metabolites that function as non-enzymatic antioxidants and membrane stabilizers [23]. Among these, terpenoids, the largest and most structurally diverse class of plant specialized metabolites, play prominent roles in abiotic stress tolerance. Volatile isoprenoids, including isoprene, monoterpenes, and sesquiterpenes, can directly scavenge ROS, quench lipid peroxidation chain reactions, and stabilize thylakoid membranes under temperature stress [24,25]. In tea plants, cold-induced sesquiterpene (nerolidol) glucosylation mediated by *UGT91Q2* enhances ROS scavenging capacity and cold tolerance, and airborne nerolidol can even trigger plant–plant communication of cold stress [26]. Cold stress also upregulates terpenoid biosynthetic genes in sandalwood and quantitatively enhances mono- and sesquiterpene emissions in tomato in proportion to stress severity [27,28]. Flavonoids, including anthocyanins, constitute another major class of cold-responsive metabolites: they accumulate in response to low temperature, scavenge ROS, and provide photoprotection [29,30], and *M. micrantha* itself accumulates anthocyanins as a visible winter adaptation [7]. Alkaloids likewise exhibit antioxidant capacity and contribute to stress defense [23]. Importantly, epigenetic regulation of secondary metabolism is now well documented: DNA methylation controls artemisinin (sesquiterpene) biosynthesis in *Artemisia annua* [31], monoterpene indole alkaloid production in Catharanthus roseus [32], and tanshinone and phenolic acid accumulation in *Salvia miltiorrhiza* [33], and epigenetic mechanisms more broadly regulate volatile organic compound emissions [34]. These findings establish that DNA methylation can act as a regulatory switch for specialized metabolic pathways, but whether such epigenetic regulation operates during cold stress in invasive plants, and whether it targets terpenoid, flavonoid, or alkaloid pathways has not been investigated.

Despite these advances, no integrated methylome–transcriptome study has examined whether cold-induced epigenetic changes reprogram secondary metabolism in invasive plants, nor has the potential dual function of such metabolic reprogramming in stress tolerance and allelopathy been addressed. Building on our previous finding that DNA methylation dynamics and antioxidant regulation accompany cold-stress germination in *M. micrantha* [22], here we employed whole-genome bisulfite sequencing and RNA-Seq of germinating embryos at 14 °C, 25 °C, and 30 °C to (i) characterize genome-wide methylation changes across CG, CHG, and CHH contexts; (ii) identify DMR-associated differentially expressed genes and their functional enrichment; and (iii) determine whether secondary metabolic pathways, particularly terpenoid biosynthesis, are subject to epigenetic regulation under cold. We hypothesized that, under low temperature, *M. micrantha* deploys epigenetic regulation to reprogram secondary metabolic networks, coordinately upregulating terpenoid biosynthesis (and potentially flavonoid and alkaloid pathways). These metabolites could potentially serve dual roles: antioxidant and membrane-protective functions that could enhance chilling tolerance, and potential accumulation of allelopathic sesquiterpene lactones that might influence competitive advantage in cold seasons, thereby potentially linking stress resistance with invasiveness. This epigenetically regulated metabolic plasticity may represent an important metabolic basis for the successful colonization of *M. micrantha* at subtropical and warm-temperate marginal habitats, and may inform predictive and management strategies for invasive plants under climate change.

## 2. Results

### 2.1. Seed Germination Pre-Experiment, RNA Extraction and Quality Assessment

**Figure 1:**
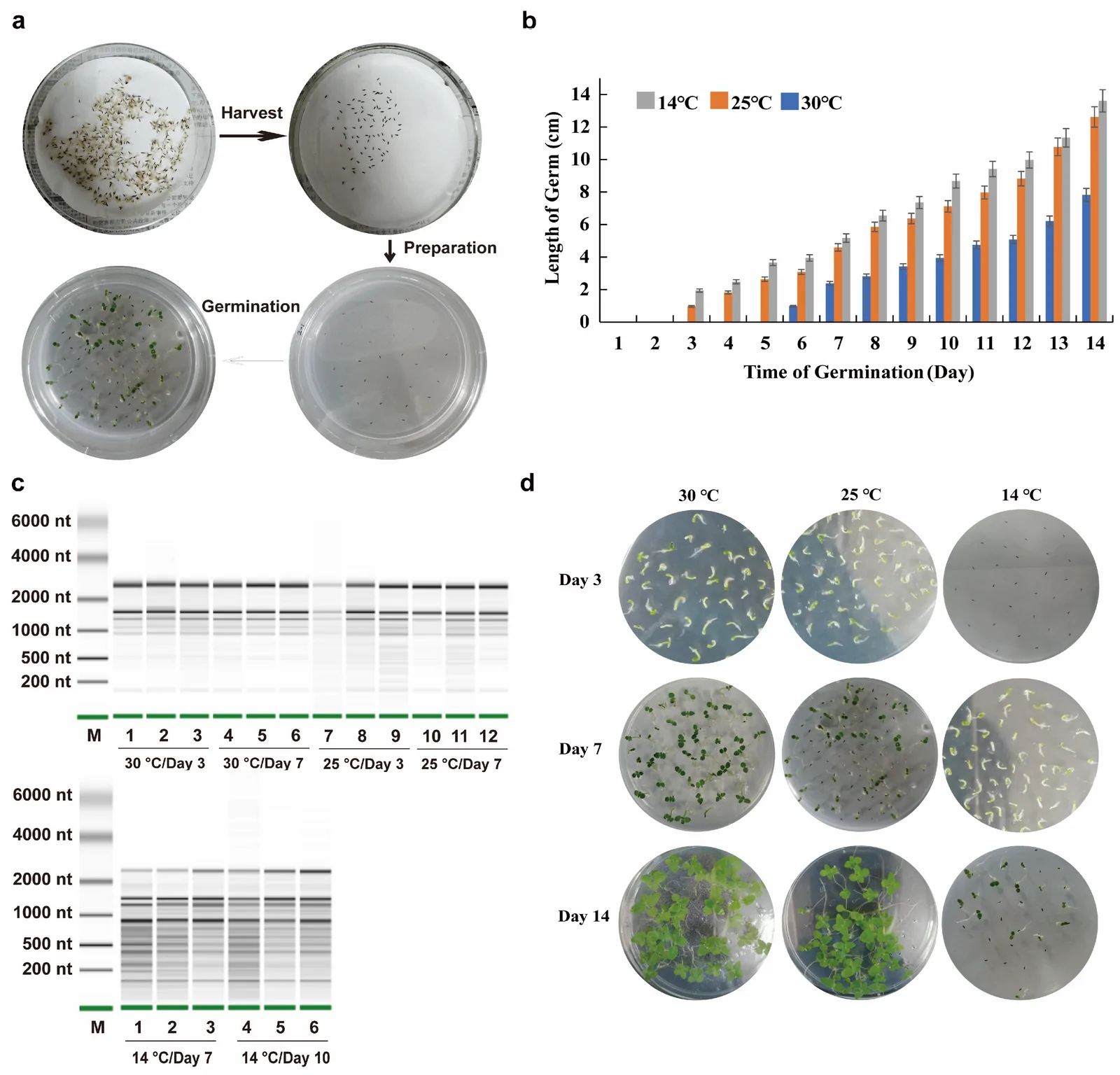
Seed germination and sample quality assessment of *M. micrantha*. (**a**) The workflow chart of *M. micrantha* seed germination; (**b**) germ length of germinating *M. micrantha* seeds; (**c**) RNA quality analysis of *M. micrantha* germs; (**d**) morphology of seedlings cultivated in MS culture medium.

Seed germination trials were performed at three constant temperatures (14 °C, 25 °C, and 30 °C) following the workflow illustrated in Figure 1a. The water uptake profiles (Supplementary Figure S1) indicated that at 30 °C, seeds underwent rapid imbibition within the first 0–18 h, reflected by a sharp increase in fresh weight, and this interval was designated imbibition phase I. After 18 h, the fresh-weight gain slowed, entering the plateau phase (protrusion phase II). Between 48 and 72 h, 40% of the seeds had ruptured the testa and successfully germinated, defining germination phase III, followed by the post-germination stage. At 25 °C, a similar pattern was observed: rapid water uptake during 0–24 h (imbibition), a plateau from 24 h to 48 h, and 40% germination occurring between 48 h and 72 h, after which post-germination commenced. Consequently, the 72 h time point (day 3) was selected as the germination milestone for both 30 °C and 25 °C.

At 14 °C, germination was substantially delayed. Imbibition was not completed until day 5, with the plateau phase extending from day 5 to day 8; germination was observed on day 9, followed by post-germination and seedling growth. According to measurements of germinating embryo length (as shown in Figure 1b), embryos at 14 °C day 7 reached a length comparable to that at 30 °C/25 °C day 3, so day 7 at 14 °C was chosen as the first sampling point. Similarly, the embryo length at 14 °C day 10 was equivalent to that at 30 °C/25 °C day 7, making day 10 at 14 °C the second sampling time. The final sampling scheme therefore comprised six time points: day 3 and day 7 at 30 °C; day 3 and day 7 at 25 °C; and day 7 and day 10 at 14 °C.

For each time point, germinating embryos of uniform, healthy appearance and free from contamination were collected. The seed coats were gently removed on ice using sterile forceps, with care taken to avoid damage to the embryos. The embryos were immediately plunged into liquid nitrogen for 2 h and subsequently stored at −80 °C. Additional embryos from the same batches were placed in RNAfixer for 48 h and then kept at −20 °C for long-term preservation. Three biological replicates were prepared per sample; the RNAfixer-preserved ones were reserved for transcriptomic profiling.

Total RNA was extracted from each sample using the RNAprep Pure Plant Kit (TIANGEN, Beijing, China). RNA concentration and integrity were evaluated using an Agilent 2100 Bioanalyzer (Agilent Technologies, Santa Clara, CA, USA) and a NanoDrop spectrophotometer (Thermo Scientific, Wilmington, DE, USA), while purity was examined via 1% agarose gel electrophoresis. The electrophoretic profiles of RNA from 18 germinating embryos of *M. micrantha* are presented in Figure 1c, and the corresponding concentration data are summarized in Table 1. Only those samples with an RNA integrity number (RIN) greater than 6.0 were retained for subsequent cDNA library construction. Distinct morphological variations were observed in seedlings cultivated on MS medium under the three temperature conditions (Figure 1d).

### 2.2. Transcriptional Reprogramming of Germinating Embryos Under Cold Stress

Using RNA-Seq technology, approximately 1.41 billion raw reads were generated across 18 libraries (six sampling time points × three biological replicates) from embryo samples collected at successive time points under 30 °C, 25 °C, and 14 °C. After quality filtration, 96.82% of the raw reads were retained, yielding approximately 1.36 billion clean reads (mean 75.8 million per library), with filtered base yields ranging from 9.86 Gb to 13.04 Gb across libraries. The overall sequencing error rate ranged from 0.02 to 0.03, while Q20 and Q30 values fell within 97.86–98.39% and 93.51–94.90%, respectively. GC contents varied between 43.77% and 47.01% across samples (Table 2). Paired-end clean reads (read 1 and read 2) were subsequently aligned to the *M. micrantha* reference genome, yielding 40,088 annotated genes with an average mapping rate of 90.31%, confirming the reliability of the transcriptomic data generated.

**Figure 2:**
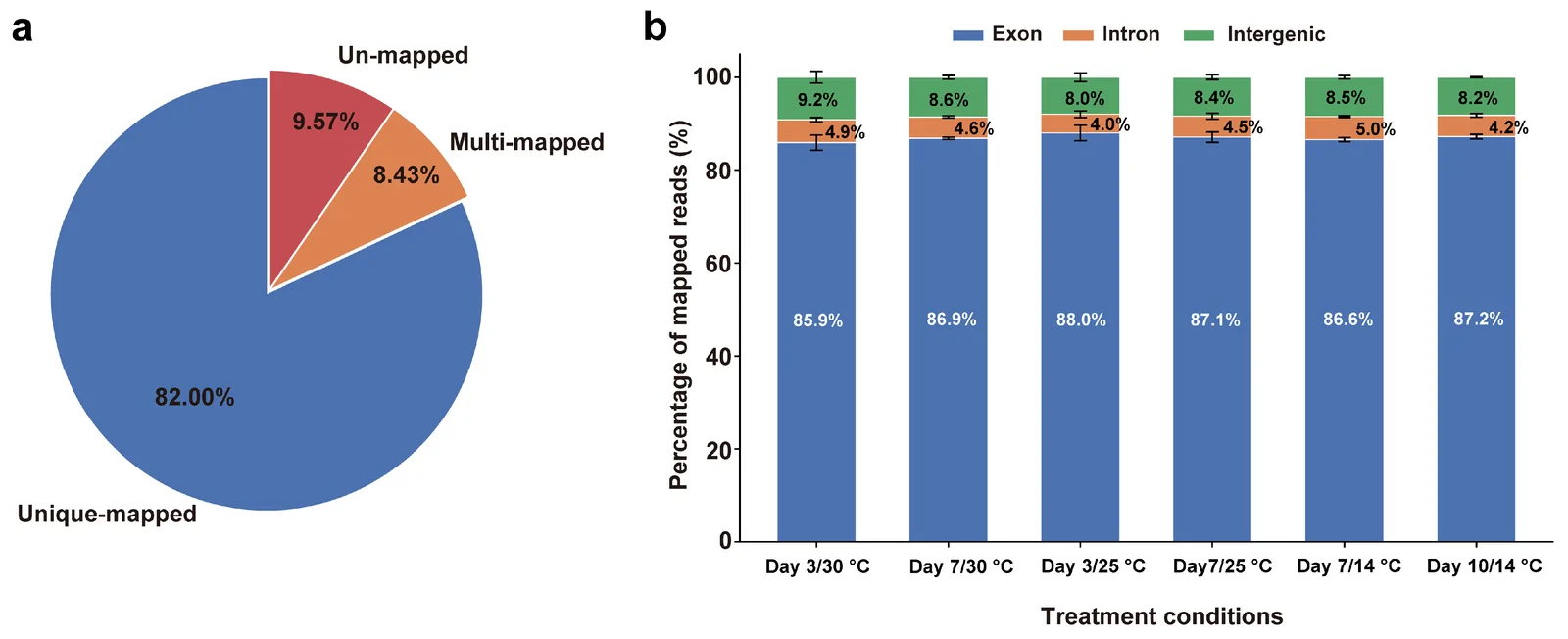
Summary of RNA-seq read mapping and genomic distribution. (**a**) Read mapping statistics of RNA-seq data; (**b**) genomic distribution of mapped reads across exonic, intronic, and intergenic regions.

These results confirm the authenticity and reliability of the transcriptome data generated by high-throughput RNA-Seq (Figure 2a). The total mapped rate (sum of read 1 and read 2 mapping rates) ranged from 74.07% to 93.14%, with uniquely mapped reads spanning 41,948,177 to 73,569,846 per sample, corresponding to unique mapping rates of 63.79% to 85.36%. Based on the alignment coordinates, reads were assigned to exonic, intronic, or intergenic regions (Figure 2b). Exonic bases accounted for 84.0–89.1% of the mapped bases, with exonic bases ranging from 6,121,371,155 to 10,643,338,959 and a maximum exonic mapping rate of 89.10% (as shown in Table 3). Intronic bases accounted for 3.48–5.39% of total alignments, likely derived from premRNA precursors or retained introns from alternative splicing events. Additionally, 7.41–10.56% of bases mapped to intergenic spaces, possibly attributable to incomplete gene annotations, noncoding RNAs, or minor DNA contamination.

### 2.3. Gene Expression Quantification and Samples Correlation Analysis

**Figure 3:**
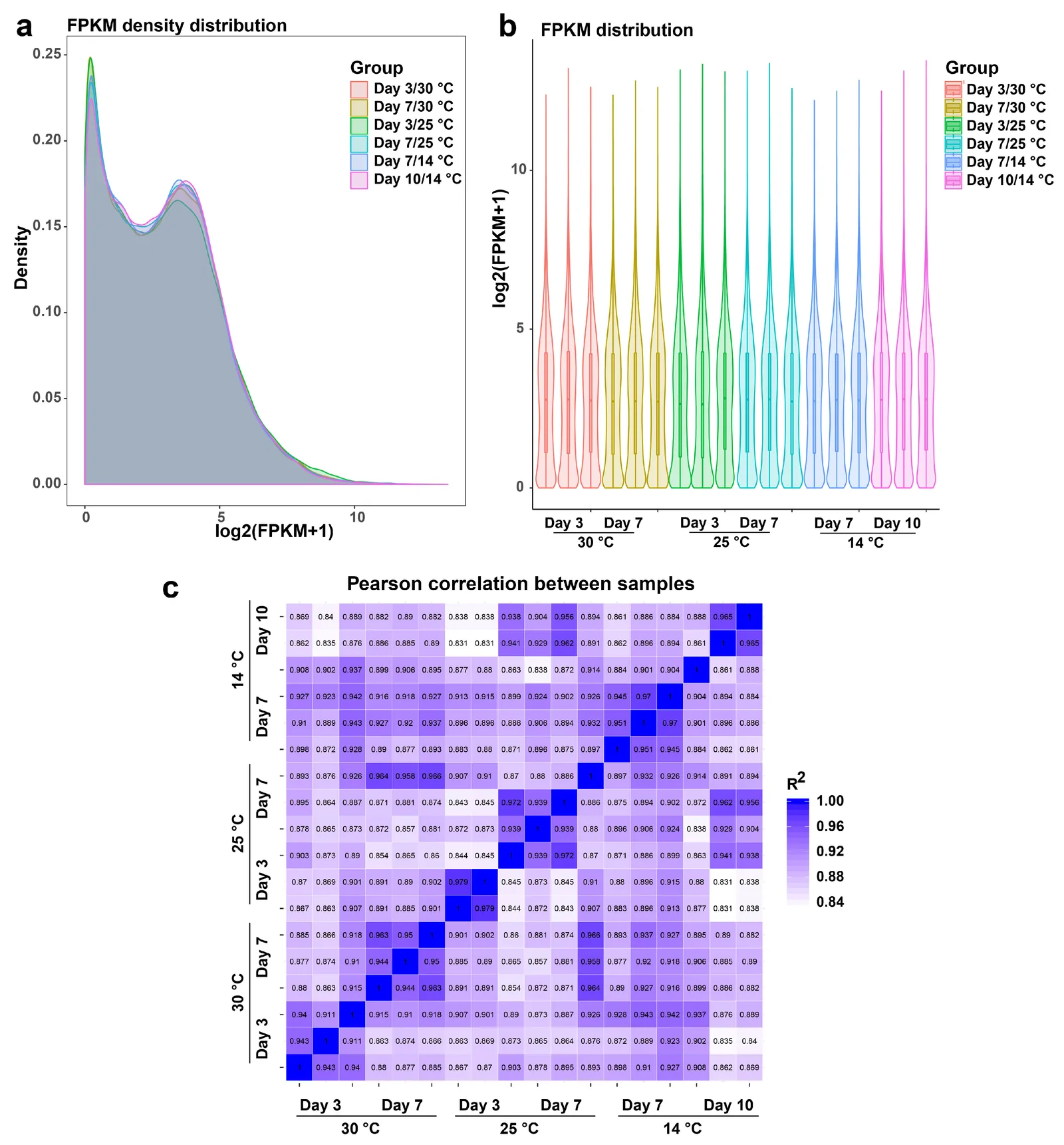
Comparative analysis of gene expression levels in *M. micrantha* germs. (**a**) The density distribution of FPKM; (**b**) FPKM-violin; (**c**) correlation analysis in biological replicates.

Gene expression levels were quantified for six sampling time points across the three temperature regimes using featureCounts (subread package) to obtain read counts, which were then converted to FPKM values. The density distribution of FPKM values across all genes is depicted in Figure 3, revealing broadly comparable patterns among the three temperature conditions. Notably, embryos germinated at 25 °C harbored a larger number of genes with low FPKM values relative to those at 30 °C and 14 °C (Figure 3a). As FPKM thresholds increased, the density of genes identified from 14 °C day 10 embryos tended to be overall higher. Violin plots further indicated that the global expression levels of genes from 14 °C-treated embryos were slightly elevated compared with those from the other two temperatures (Figure 3b).

To assess consistency among biological replicates, Pearson’s correlation coefficients were computed based on gene expression profiles (Figure 3c). Replicate groups exhibited high reproducibility, fulfilling the prerequisites for subsequent differential expression analyses. Specifically, the correlation coefficients exceeded 0.9 for replicates at 30 °C day 3, 30 °C day 7, and 14 °C day 7, while those at 25 °C day 7 and 14 °C day 10 were greater than 0.8, confirming the reliability of the expression data.

### 2.4. Differential Gene Expression Analysis and Functional Enrichment

**Figure 4:**
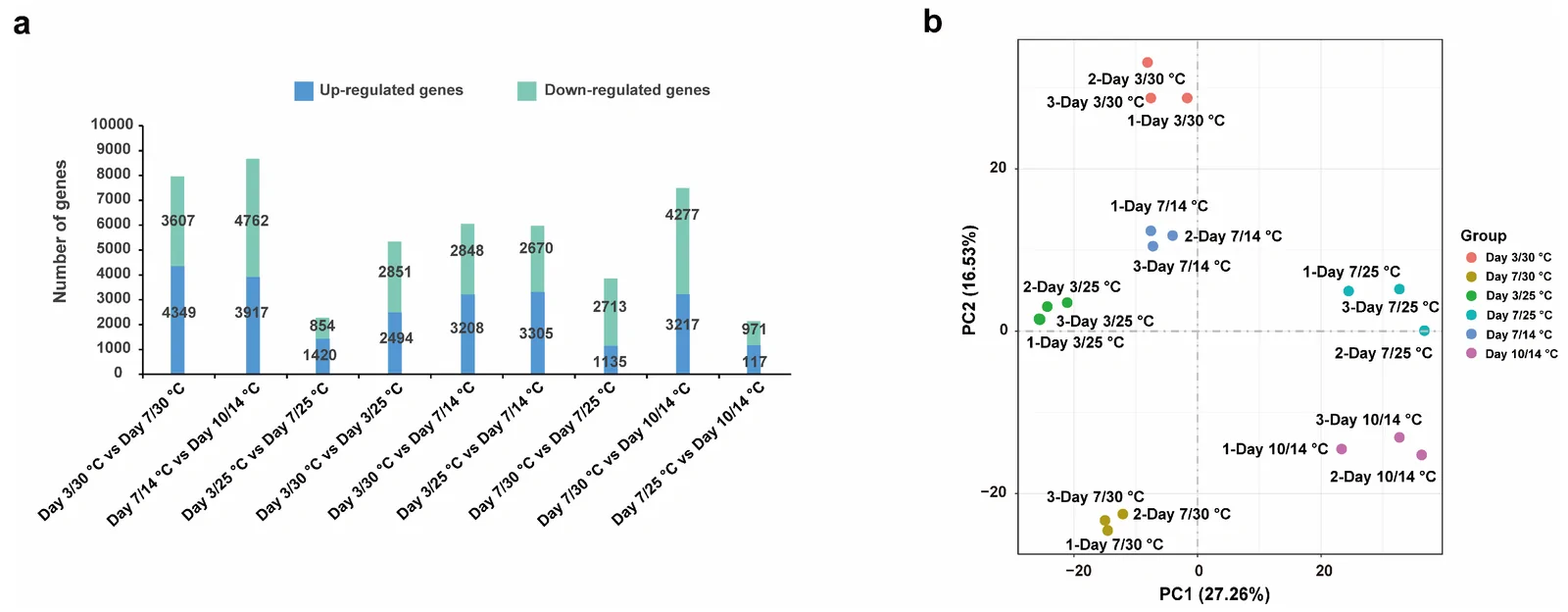
Differentially expressed genes and their PCA. (**a**) Number of differentially expressed genes among three temperatures; (**b**) principal component analysis of DEGs.

Pairwise comparisons of gene expression were performed between embryos germinated at the same temperature but at two germination time points, and between embryos germinated at different temperatures but at a comparable germ length, yielding nine pairwise comparisons in total, as shown in Figure 4a. Considerable transcriptional changes occurred across successive developmental stages under each temperature. At 30 °C, 7956 differentially expressed genes (DEGs) were identified between day 3 and day 7, of which 4349 were upregulated and 3607 downregulated. At 25 °C, the corresponding comparison yielded 2274 DEGs (1420 upregulated and 854 downregulated). The largest number of DEGs was observed at 14 °C between day 7 and day 10, with 8679 DEGs (3917 upregulated and 4762 downregulated). Furthermore, during the early germination phase, 5975 DEGs were detected between 25 °C and 14 °C, but this number decreased to 1088 at later stages. Principal component analysis (PCA) showed good clustering of biological replicates, with PC1 and PC2 accounting for 27.26% and 16.53% of the total variance, respectively (Figure 4b).

### 2.5. Quality Control of Bisulfite Sequencing Data

DNA concentrations were determined using a Qubit 2.0 Fluorometer; all samples exhibited acceptable DNA integrity and purity, with concentrations ranging from 3.58 ng/μL to 14.08 ng/μL. The genome coverage distribution across all samples, where the abscissa indicates coverage depth and the ordinate represents the proportion of genomic bases covered at a given depth. At the genome-wide level, when coverage was below 20×, a higher proportion of bases was observed in embryos germinated at 14 °C; conversely, at coverage depths above 20×, samples from 25 °C exhibited a greater proportion of covered bases. Genome coverage statistics are summarized in Table 4. The total number of detected sites ranged from 1,343,423,094 to 1,369,305,764 across samples. The mean coverage depth over all genomic sites ranged from 13.14× to 16.76×. The fraction of bases with ≥1× sequencing depth ranged from 87.64% to 89.33% of the total genome, while those with ≥5× and ≥10× coverage accounted for 73.42–77.79% and 56.65–64.56%, respectively, indicating that at least 56.65% of cytosine sites were covered at ≥10× sequencing depth.

Coverage statistics for cytosine sites are detailed in Table 5. The mean coverage depth across all C sites ranged from 5.7 to 7.3. The number of aligned bases on genomic C positions varied between 3157.5 Mb and 4064.5 Mb; for cytosines in the CG context, the aligned bases ranged from 411.2 Mb to 528.8 Mb; in the CHG context, from 411.7 Mb to 528.7 Mb; and in the CHH context, from 2334.5 Mb to 3007 Mb. The overall average methylation level across all genomic C sites ranged from 23.55% to 25.54%. Among the three sequence contexts, CG exhibited the highest average methylation level (80.21–84.63%), followed by CHG (63.67–66.77%), while CHH displayed the lowest average methylation level, ranging from 6.63% to 7.51%.

### 2.6. Integrated Epigenetic and Transcriptomic Profiling

**Figure 5:**
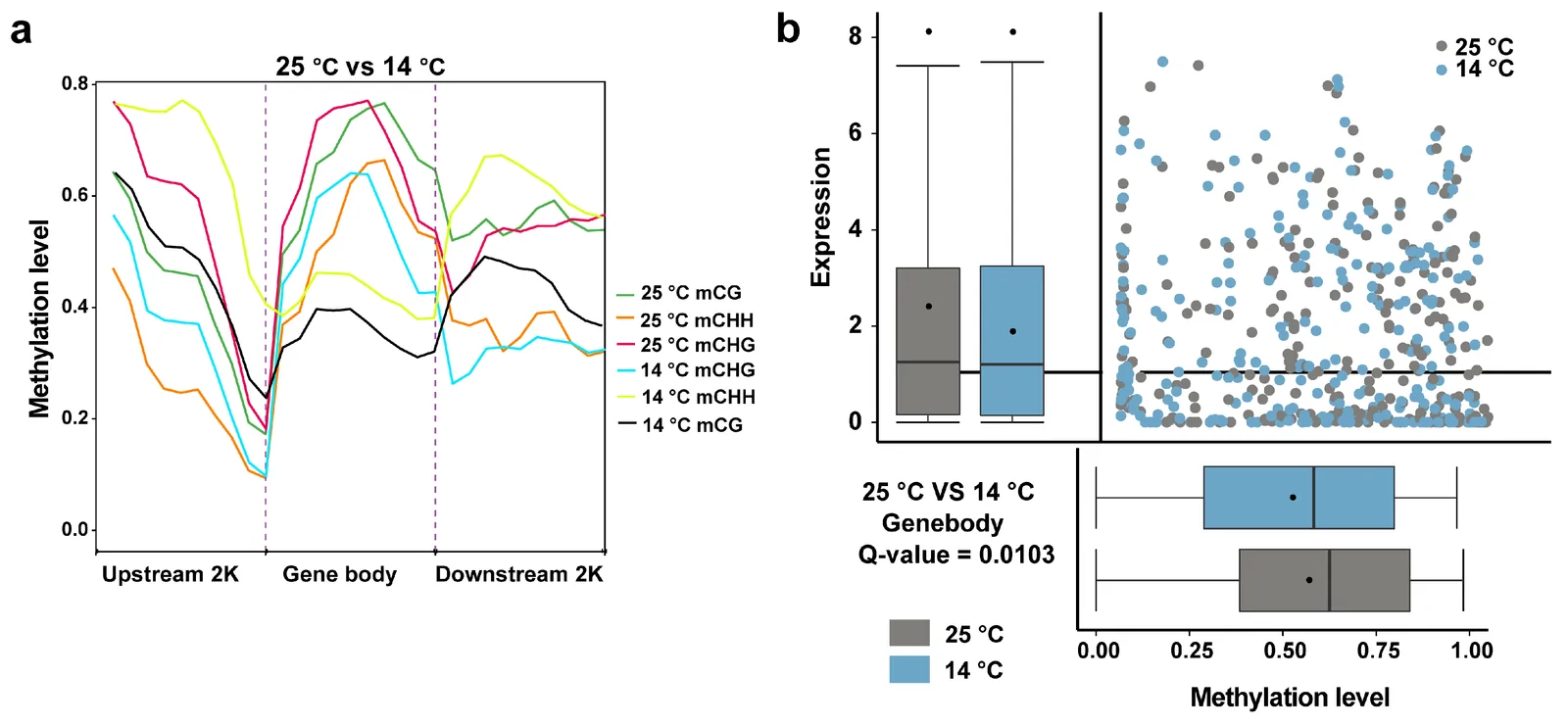
Analysis of the DNA methylation level under different contexts (25 °C vs. 14 °C). (**a**) Regional methylation levels in three sequence contexts of *M. micrantha* germs across gene-body and flanking regions in samples (25 °C and 14 °C); (**b**) expression and methylation levels of samples across gene body.

For a more detailed assessment of the dynamic interplay between DNA methylation and transcriptional activity during embryo development, methylation levels at individual genes were directly compared with their corresponding expression levels. As depicted in Figure 5a, flanking regions of genes exhibited elevated DNA methylation levels in the CHH context in embryos germinated at 14 °C.

Within the gene body, methylation levels in the CG and CHG contexts were slightly higher in embryos germinated at 25 °C. Overall, comparative analysis of methylomes and transcriptomes between embryos germinated at 25 °C and 14 °C revealed the same trend between gene-body methylation and gene expression (Figure 5b). Overall, comparative methylome and transcriptome analyses of embryos germinated at 25 °C and 14 °C revealed concordant changes in gene-body methylation and gene expression (Figure 5b).

### 2.7. Functional Annotation Analysis of DMR-Related Genes

**Figure 6:**
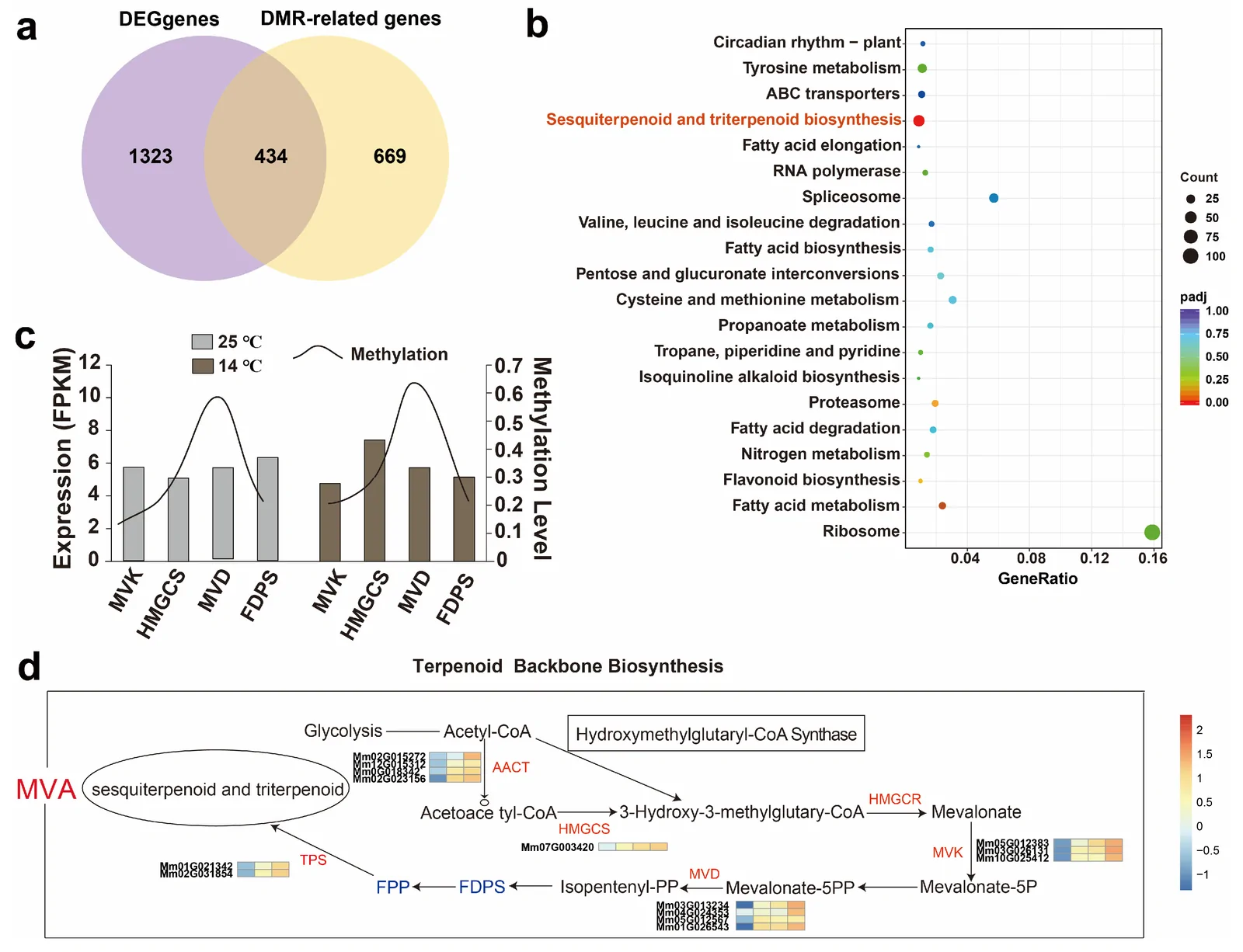
Analysis of DEGs and DMR-related genes identified from *M. micrantha* germs. (**a**) Numbers of differentially expressed genes and differentially methylated region-related genes; (**b**) KEGG enrichment bubble map of overlapped genes; (**c**) changing tendency between methylation level and FPKM of genes; (**d**) the matched DEGs involved in sesquiterpenoid and triterpenoid backbone biosynthesis among embryos at 25 °C and 14 °C.

To investigate the relationship between DNA methylation and gene expression, the expression profiles of genes associated with differentially methylated regions (DMRs) were examined across distinct seed developmental stages using RNA-Seq data. In the comparison between embryos germinated at 25 °C and 14 °C, a total of 1757 differentially expressed genes (DEGs) and 1103 DMR-associated genes were identified, with 434 genes overlapping between the two sets (Figure 6a). KEGG functional enrichment analysis of the 434 overlapping genes revealed that the sesquiterpenoid and triterpenoid biosynthesis pathways were significantly enriched, containing a substantial number of genes (Figure 6b). We subsequently performed an integrated analysis of transcript abundance and DNA methylation levels for four expressed genes (*MVK*, *HMGCS*, *MVD*, and *FDPS*) involved in this pathway. As shown in Figure 6c, the relationship between expression and DNA methylation varied among these genes in *M. micrantha* embryos germinated at 25 °C and 14 °C; *HMGCS* showed clearly higher FPKM at 14 °C, whereas *MVK* and *FDPS* appeared lower and *MVD* remained largely unchanged.

Based on the significant enrichment of DEGs in sesquiterpenoid and triterpenoid biosynthesis pathways identified by KEGG analysis, we conducted a systematic investigation into terpenoid backbone biosynthesis by the cytosolic mevalonate (MVA) pathway. As shown in Figure 6d, acetyl-CoA was first condensed to acetoacetyl-CoA by acetoacetyl-CoA thiolase (*AACT*), and *HMGCS* subsequently catalyzes the formation of 3-hydroxy-3-methylglutaryl-CoA (HMG-CoA). *HMGCR* then reduces HMG-CoA to mevalonate, which is sequentially phosphorylated by *MVK* and *MVD* to yield IPP. *FDPS* further condenses IPP with DMAPP to generate FPP, the committed precursor for sesquiterpenoid and triterpenoid biosynthesis (distinct from GPP, which feeds monoterpenoid biosynthesis), and FPP is ultimately channeled to sesquiterpenoids and triterpenoids through *TPS*. Among these genes, *HMGCS* showed clearly higher expression at 14 °C, whereas *MVK* and *FDPS* showed lower FPKM, and *MVD* remained largely unchanged (Figure 6c).

## 3. Discussion

Environmental factors are known to modulate epigenetic traits and adaptive evolutionary processes in plants [35]. Cytosine methylation (5-mC) serves as an epigenetic mark associated with both programmed development and stress responses, and DNA methylation has been demonstrated to regulate plant responses to environmental challenges [15,36]. To elucidate the epigenetic regulatory mechanisms underlying cold adaptation during seed germination in *M. micrantha*, we analyzed genome-wide DNA methylation dynamics in embryos germinated under control conditions (25 °C and 30 °C) and cold treatment (14 °C).

Epigenetic modifications, exemplified by DNA methylation, play critical roles in the regulation of genome stability, genomic imprinting, transposon silencing, and gene expression during plant development and stress defense [37,38]. In animals, DNA methylation occurs predominantly in the CG sequence context, whereas in plants, it is commonly found in CG, CHG, and CHH contexts [35]. Previous studies have established that methylation patterns generated within a given species are relatively stable and can be inherited across generations, which to some extent reflects the genetic stability of the species [36]. Upon exposure to stress, however, genomic DNA methylation in plants can undergo alterations, and this has been considered as one of the adaptive mechanisms in response to adverse environments [15,39]. In conjunction with the methylation and expression profiles observed in *M. micrantha* embryos germinated at low temperature, it is plausible that this species enhances its cold tolerance by increasing the expression of stress-responsive genes under chilling conditions. In addition, the expression of genes encoding antioxidant enzymes may also be subject to regulation by DNA methylation.

Beyond antioxidant enzymes, terpenoid metabolism represents an additional layer of plant defense against cold-induced oxidative stress. Accumulating evidence indicates that cold stress can actively trigger terpenoid accumulation in plants [26]. These specialized metabolites possess intrinsic antioxidant activity and can directly scavenge excess reactive oxygen species (ROS) generated under low-temperature conditions, thereby alleviating membrane lipid peroxidation and preserving cellular membrane integrity [24,27]. In agreement with this notion, our integrated methylome and transcriptome analysis revealed significant enrichment of the sesquiterpenoid and triterpenoid biosynthesis pathways, with *HMGCS* showing clearly higher expression in embryos germinated at 14 °C, whereas the other MVA pathway genes examined showed either unchanged or lower expression. The temperature-dependent expression of these genes suggests that altered terpenoid biosynthesis may constitute an epigenetically regulated adaptive strategy, whereby *M. micrantha* mitigates cold-induced oxidative damage through increased production of antioxidant terpenoids. We note, however, that these inferences rest on methylome and transcriptome data alone; terpenoid metabolites were not directly quantified in this study, and the proposed protective roles therefore remain to be confirmed by GC-MS/LC-MS metabolite profiling and functional assays.

The regulation of gene expression by DNA methylation does not occur in isolation, but rather in coordination with multiple epigenetic mechanisms, including histone modifications, chromatin remodeling, and non-coding RNAs, collectively constituting a complex regulatory network [11,40]. During plant responses to low-temperature stress, dynamic changes in DNA methylation are often accompanied by reprogramming of histone H3 lysine 9 and lysine 27 methylation, as well as the involvement of small RNA pathways, which together fine-tune the spatiotemporal expression patterns of stress-responsive genes [10,41]. Notably, as a globally invasive weed, *M. micrantha* may owe its remarkable environmental adaptability, at least in part, to the flexibility and plasticity of its epigenetic regulation [21]. The functional mechanisms underlying the observed CHH hypermethylation in flanking regions and its relationship to transcriptional activity remain to be fully elucidated. Future investigations should integrate chromatin state analysis, transcription factor binding site prediction, and locus-specific bisulfite sequencing to further dissect the relationship between flanking CHH methylation and gene expression in the cold adaptation of *M. micrantha*. In addition, given the high sensitivity of epigenetic modifications to environmental cues, subsequent studies should pay greater attention to the epigenomic changes in *M. micrantha* under diverse environmental stresses, such as drought, salinity, and high temperature, in order to comprehensively evaluate the contribution of epigenetic regulation to the widespread invasiveness of this species.

Although the RNA-Seq data derive from the same experiment as our earlier work [22], which examined antioxidant enzyme regulation during cold-stress germination in *M. micrantha*, the present study provides substantially new analyses that were not included in the previous report: a genome-wide WGBS methylome across CG, CHG, and CHH contexts, genome-wide DMR identification and annotation, integrative methylome–transcriptome analysis of epigenetically regulated DEGs, and the epigenetic characterization of terpenoid biosynthesis pathway regulation. These new data extend our earlier observation that cold-induced demethylation accompanies antioxidant responses by identifying a second, metabolically distinct layer of epigenetic reprogramming under chilling stress.

Two limitations of this study should be acknowledged. First, although sampling was matched by embryo length to compare developmentally equivalent stages, the comparison of 14 °C day 7 with 25 °C day 3 (and 14 °C day 10 with 25 °C day 7) cannot fully separate temperature effects from chronological-age-dependent epigenetic changes; higher-resolution time-series sampling or synchronized developmental systems will be required to disentangle them. Second, this is a correlative study without functional validation: conclusions about epigenetic regulation of terpenoid biosynthesis remain tentative and require qRT-PCR validation of DEGs, locus-specific bisulfite validation of DMRs, GC-MS/LC-MS terpenoid quantification, and allelopathy bioassays in future work.

## 4. Materials and Methods

### 4.1. Plant Material and Seed Germination Assay

Mature seeds were collected from *M. micrantha* grown on Neilingding Island (Shenzhen, Guangdong, China). After harvest, the seeds were stored at −20 °C until their dormancy was completely released. Surface sterilization was carried out by immersing the seeds in 75% ethanol for 5 min, followed by five washes with sterile deionized water, and then treating with 20% sodium hypochlorite solution for 30 min, again rinsing five times with sterile deionized water. Each batch of 30 seeds was dried on sterilized filter paper and placed in 90-mm Petri dishes containing Murashige and Skoog (MS) medium composed of 0.443% MS salts, 3% sucrose, and 0.75% agar. The seeds were incubated in a growth chamber under three temperature regimes (14 °C, 25 °C, and 30 °C) with a photosynthetic photon flux density of 100 μmol·m^−2^·s^−1^, a 16 h light and 8 h dark photoperiod, and 40–60% relative humidity for two weeks. Germination was recorded when the radicle protruded through the seed coats and reached 0.5 cm in length; counts were performed daily over the 14-day period. For soil-grown experiments, the same environmental conditions were used. Three hundred seeds were sown in three pots (100 seeds per pot) filled with a substrate mixture of soil and vermiculite (1:1, *v*/*v*).

Samples were collected on days 3 and 7 after imbibition for the 25 °C and 30 °C treatments, and on days 7 and 10 for the 14 °C treatment. After gentle washing with sterile water, the germinating seeds (day 3 at 25 °C and 30 °C, day 7 at 14 °C) were dissected on ice to remove the seed coats. The other portion was preserved in RNAfixer (BioTeke, Shanghai, China) at −20 °C for later RNA-Seq analysis.

### 4.2. RNA Isolation, Illumina Sequencing and Differential Gene Analysis

Total RNA was extracted using the TIANGEN RNAprep Pure Plant Kit (TIANGEN, Beijing, China). RNA concentration and integrity were assessed with an Agilent 2100 Bioanalyzer (Agilent Technologies, Santa Clara, CA, USA) and a NanoDrop spectrophotometer (Thermo Scientific, Wilmington, DE, USA), while purity was examined by 1% agarose gel electrophoresis. Only samples with RIN > 6.0 were used for cDNA library construction; this threshold was selected because plant seed tissues are rich in polyphenols and polysaccharides that can depress Bioanalyzer-based RIN values even when the RNA remains intact, as additionally verified by agarose gel electrophoresis and the high sequencing mapping rates (mean 90.31%). Libraries were prepared following the NEBNext Ultra RNA Library Prep Kit protocol (New England Biolabs, Ipswich, MA, USA). Poly(A) mRNAs were enriched using oligo(dT)-coated magnetic beads, followed by rRNA removal. The mRNAs were then fragmented, and first-strand and second-strand cDNAs were synthesized. After end repair, A-tailing, and adapter ligation, cDNA fragments of 250–300 bp were selected with AMPure XP beads (Beckman Coulter, Brea, CA, USA), PCR-amplified, and purified. Libraries were initially quantified with a Qubit 2.0 Fluorometer (Thermo Fisher Scientific, Waltham, MA, USA), diluted to 1.5 ng/μL, and insert sizes were verified on an Agilent 2100 Bioanalyzer. Library concentrations were accurately determined by qRT-PCR. Qualified libraries were pooled and sequenced on an Illumina NovaSeq 6000 platform (Illumina, San Diego, CA, USA) (paired-end 150 bp).

Clean reads were aligned to the *M. micrantha* reference genome using HISAT2 (v2.2.1; Johns Hopkins University, Baltimore, MD, USA), and gene expression levels were calculated as FPKM; gene-level read counts were obtained with featureCounts (Subread package, v2.0.1; Walter and Eliza Hall Institute, Melbourne, Australia). Expression distributions across samples were visualized by box plots (Python), and sample correlations were displayed as heatmaps using the R package gplot (v3.1.3; R Foundation for Statistical Computing, Vienna, Austria), with R^2^ > 0.8 indicating similar expression patterns. For each group, the average FPKM of three biological replicates was used for visualization and correlation analysis only. Differentially expressed genes (DEGs) were identified using DESeq2 (v1.30.1) with an adjusted *p*-value (FDR) < 0.05 and |log_2_(Fold Change)| ≥ 1, and further classified as up- or down-regulated. Clustering and functional enrichment analyses were then performed on the DEGs. The RNA-Seq data originated from the same experiment reported in our previous study [22], which focused on antioxidant enzyme genes; the present study extends the analysis to genome-wide methylation profiling, DMR identification, methylome–transcriptome integration, and terpenoid biosynthesis pathway regulation that were not addressed previously.

### 4.3. Bisulfite Sequencing Data Processing, Methylome Characterization, and Differential Methylation Analysis

Genomic DNA was extracted from germinating embryos using the CTAB method, and DNA concentration and integrity were assessed with a Qubit 2.0 Fluorometer(Thermo Fisher Scientific, Waltham, MA, USA)and 1% agarose gel electrophoresis. For whole-genome bisulfite sequencing (WGBS), 1 μg of qualified genomic DNA was fragmented to 200–300 bp by sonication, end-repaired, 3′-adenylated, and ligated to methylated-cytosine adapters. Bisulfite conversion was performed using the EZ DNA Methylation-Gold Kit (Zymo Research, Irvine, CA, USA) following the manufacturer’s instructions, and unmethylated lambda DNA (Promega, Madison, WI, USA) was spiked in as a conversion control. Converted DNA was PCR-amplified, purified, and size-selected to construct the WGBS libraries, which were sequenced on an IlluminaNovaSeq 6000 platform (Illumina, San Diego, CA, USA) (paired-end 150 bp).

Bismark (v0.16.3) with default parameters was used for reference genome alignment of bisulfite-sequencing data, using the *M. micrantha* genome annotation as reference. The genome and reads were converted in silico (C-to-T and G-to-A) prior to alignment, and only uniquely paired-end mapped reads were retained. Methylated cytosine (mC) calling and coverage statistics were performed using Bismark (v0.16.3). Duplicate reads from PCR were removed. A cytosine was considered methylated if aligned as C, and unmethylated if aligned as T. Bisulfite conversion efficiency was assessed using spiked-in unmethylated lambda DNA and was ≥ 99.2% for every library. Binomial tests were applied to each C site with Q value < 0.01 to determine true methylation status. Sites with sequencing depth > 5× were retained for downstream analysis. We calculated the number of mC sites, average coverage per site, and proportions of bases with depth > 1×, >5×, and >10× relative to the genome size. Chromosome-level read depth and coverage were also assessed.

Three sequence contexts (CG, CHG, CHH; H = A, T, or C) were analyzed. For each context, we calculated the number of aligned C bases, the proportion of methylated C sites out of total C sites (mC percent, mCpG percent, mCHG percent, mCHH percent), and average coverage depth. The methylation level (ML) for each mC site was defined as ML = mC/(mC + umC), where mC = reads supporting methylation and umC = reads supporting unmethylation. Single-sample methylation analysis included density statistics at genome-wide and chromosome levels, distributions across genomic functional regions (promoter, exon, intron, repeat region, 2 kb upstream of transcription start site (TSS), and 2 kb downstream of transcription termination site (TES), and average methylation levels in genebody, TSS-upstream, and TES-downstream regions. Sample correlation was assessed by Pearson’s correlation coefficient based on methylation levels in 2 kb bins.

Differentially methylated regions (DMRs) were identified using DSS (v2.12.0), considering distance dependency, read depth, and biological replicate heterogeneity, applying the following criteria: minimum read depth ≥ 5×, at least three cytosine sites within each DMR, minimum DMR length ≥ 100 bp, methylation-level difference ≥ 0.2, and FDR-adjusted *p*-value < 0.05. Violin and Circos plots were used to visualize methylation levels and global differences between comparison groups. DMRs were classified into hyper- and hypo-methylated regions across functional elements (promoter, exon, intron, repeat region, TSS, TES). DMR-anchored genes were defined as those with at least 1 bp overlap between the DMR and the gene body (from TSS to TES). Functional annotation of these genes was performed usingGPseq (v1.0; Beijing Genomics Institute, Shenzhen, China)for GO enrichment (Q value < 0.05) andKOBAS (v3.0; Peking University, Beijing, China)for KEGG pathway analysis.

### 4.4. Integrated Analysis of DNA Methylation and Transcriptome

Raw DNA methylation data and RNA-sequencing data were preprocessed separately using standard pipelines. For the WGBS data, bisulfite reads were aligned with Bismark (v0.16.3) and DMRs were identified with DSS (v2.12.0). Differentially expressed genes (DEGs) were called from RNA-seq data using DESeq2 (v1.30.1) (|log_2_FC| ≥ 1, adjusted *p*-value < 0.05). All statistical thresholds were adjusted for multiple testing using the Benjamini–Hochberg false discovery rate (FDR) method.

The DMR-mapped genes were intersected with the DEG list using Venn diagrams, and the overlapping genes, defined as epigenetically regulated DEGs, were carried forward for functional and regulatory analyses. Enrichment of DEGs was performed using KEGG pathways via clusterProfiler (4.12.5), with FDR < 0.05 as the significance cutoff. Furthermore, average methylation levels in gene-body, upstream, and downstream regions were compared with gene expression to assess region- and context-dependent associations.

### 4.5. Data Analysis

Statistical analyses were performed using R (v4.0.3) and Python (v3.8). Differential expression and differential methylation analyses were conducted with DESeq2 and DSS, respectively, and an FDR-adjusted *p*-value < 0.05 was considered significant. Principal components analysis (PCA) was conducted using the prcomp function in R.

## 5. Conclusions

Integrative methylomic and transcriptomic profiling of germinating *M. micrantha* embryos at 14 °C, 25 °C, and 30 °C suggests that cold stress delays but does not arrest germination and is accompanied by context- and position-dependent DNA methylation reprogramming. The 2 kb upstream (promoter) and downstream flanking regions exhibited markedly elevated CHH methylation under cold (14 °C) germination, whereas gene-body methylation—particularly in the CG and CHG contexts—was slightly higher at 25 °C and showed an overall positive association with gene expression. Among 434 DMR-associated differentially expressed genes, the sesquiterpenoid and triterpenoid biosynthesis pathway is significantly enriched, with key MVA pathway genes (*AACT*, *HMGCS*, *MVD*, *MVK*, *FDPS*) showing differential expression associated with DMRs at 14 °C. These findings are consistent with the hypothesis that cold-associated epigenetic changes are linked to transcriptional regulation of terpenoid biosynthesis, which may contribute to chilling tolerance through antioxidant and membrane-protective terpenoids; the possible production of allelopathic sesquiterpene lactones and its ecological consequences remain untested working hypotheses. This potential link of stress tolerance with invasion chemistry via epigenetic regulation may represent an important metabolic basis for the colonization of *M. micrantha* at subtropical and warm-temperate marginal habitats, and offers new perspectives for invasive plant management under climate change, while functional validation is required to establish causal epigenetic control.

## Figures and Tables

**Table 1:** Quality test of total RNA.

Samples		Concentration (ng/μL)	Volume (μL)	Total Mass (μg)	RIN
30 °C/Day 3	Repeat 1	344	35	12.04	7.4
	Repeat 2	113	35	3.955	7.3
	Repeat 3	278	35	9.73	6.7
30 °C/Day 7	Repeat 1	335	35	11.725	6.9
	Repeat 2	181	35	6.335	7.4
	Repeat 3	246	35	8.61	7.2
25 °C/Day 3	Repeat 1	28	35	0.98	7.5
	Repeat 2	186	35	6.51	6.6
	Repeat 3	345	35	12.075	6.2
25 °C/Day 7	Repeat 1	273	35	9.555	7.8
	Repeat 2	527	35	18.445	6.4
	Repeat 3	403	35	14.105	7.1
14 °C/Day 7	Repeat 1	649	35	22.715	7.7
	Repeat 2	361	35	12.635	7.0
	Repeat 3	310	35	10.85	7.4
14 °C/Day 10	Repeat 1	212	35	7.42	7.8
	Repeat 2	175	35	6.125	7.5
	Repeat 3	175	35	6.125	7.2

**Table 2:** Quality test of raw reads.

Samples		Raw Reads	Clean Reads	Clean Bases (G)	Error Rate	Q20	Q30	GC
30 °C/Day 3	Repeat 1	75,829,628	73,320,932	11.0	0.02	98.22	94.61	44.40
	Repeat 2	67,901,402	65,763,912	9.86	0.02	98.26	94.73	47.01
	Repeat 3	74,149,476	71,675,696	10.75	0.02	98.22	94.57	43.97
30 °C/Day 7	Repeat 1	74,025,058	72,666,690	10.9	0.02	98.39	94.90	43.89
	Repeat 2	80,683,650	78,515,638	11.78	0.02	98.21	94.62	44.39
	Repeat 3	76,118,756	73,711,924	11.06	0.02	98.24	94.59	44.21
25 °C/Day 3	Repeat 1	79,123,748	76,316,492	11.45	0.02	98.18	94.26	43.77
	Repeat 2	91,305,672	86,923,634	13.04	0.02	98.14	94.36	44.20
	Repeat 3	77,100,066	74,703,132	11.21	0.02	98.37	94.82	43.90
25 °C/Day 7	Repeat 1	82,860,540	81,568,370	12.24	0.02	98.36	94.71	44.19
	Repeat 2	75,402,466	72,866,188	10.93	0.03	97.91	93.70	44.05
	Repeat 3	78,619,892	76,063,302	11.41	0.03	97.88	93.64	43.93
14 °C/Day 7	Repeat 1	85,073,624	81,290,004	12.19	0.03	97.86	93.51	44.05
	Repeat 2	79,684,276	77,309,070	11.6	0.03	97.88	93.67	43.91
	Repeat 3	78,498,664	76,392,316	11.46	0.03	97.91	93.73	44.48
14 °C/Day 10	Repeat 1	76,347,612	73,845,590	11.08	0.03	97.97	93.84	43.98
	Repeat 2	79,208,850	76,641,508	11.5	0.03	97.93	93.77	43.89
	Repeat 3	77,025,284	74,609,926	11.19	0.03	97.98	93.80	44.00

**Table 3:** Analysis of genome region distribution.

Samples		Exon Bases (Percentage)	Intron Bases (Percentage)	Intergenic Bases (Percentage)
Day 3/30 °C	Repeat 1	8,771,851,322 (86.7672%)	450,163,057 (4.4528%)	887,627,006 (8.78%)
	Repeat 2	6,121,371,155 (84.0443%)	392,630,092 (5.3907%)	769,506,159 (10.565%)
	Repeat 3	8,611,252,709 (86.9791%)	485,277,300 (4.9016%)	803,836,879 (8.1193%)
Day 7/30 °C	Repeat 1	8,779,500,208 (86.9066%)	487,357,897 (4.8243%)	835,358,804 (8.2691%)
	Repeat 2	9,398,691,196 (86.6121%)	475,365,533 (4.3807%)	977,416,947 (9.0072%)
	Repeat 3	8,898,381,518 (87.0581%)	461,434,960 (4.5145%)	861,383,354 (8.4274%)
Day 3/25 °C	Repeat 1	9,343,953,223 (88.8064%)	394,268,798 (3.7472%)	783,488,884 (7.4464%)
	Repeat 2	10,643,338,959 (89.1049%)	415,947,636 (3.4823%)	885,433,345 (7.4128%)
	Repeat 3	8,920,776,099 (86.0522%)	509,047,887 (4.9104%)	936,882,045 (9.0374%)
Day 7/25 °C	Repeat 1	9,804,039,197 (86.2729%)	581,381,769 (5.116%)	978,570,085 (8.6111%)
	Repeat 2	8,735,318,655 (86.6865%)	460,508,154 (4.5699%)	881,084,402 (8.7436%)
	Repeat 3	8,631,890,128 (88.3461%)	376,887,498 (3.8574%)	761,760,456 (7.7965%)
Day 7/14 °C	Repeat 1	9,591,390,817 (86.1115%)	572,445,593 (5.1394%)	974,502,042 (8.7491%)
	Repeat 2	9,270,867,344 (86.9748%)	528,181,956 (4.9552%)	860,197,440 (8.07%)
	Repeat 3	9,142,457,461 (86.662%)	503,891,989 (4.7764%)	903,208,962 (8.5616%)
Day 10/14 °C	Repeat 1	8,813,284,690 (86.6691%)	512,450,398 (5.0394%)	843,154,826 (8.2915%)
	Repeat 2	8,146,787,775 (87.3128%)	415,592,105 (4.4541%)	768,198,542 (8.2331%)
	Repeat 3	8,851,688,065 (87.6285%)	438,525,923 (4.3412%)	811,164,965 (8.0302%)

**Table 4:** Summary of genome coverage.

Samples		Site Number	Site covgMean	Sites numCovg1	Sites numCovg2	Sites numCovg3
30 °C/Day 3	Repeat 1	1,361,051,927	14.65	88.79	76.00	60.96
	Repeat 2	1,353,500,192	13.86	88.29	74.71	58.89
	Repeat 3	1,360,432,239	15.50	88.75	76.33	62.22
30 °C/Day 7	Repeat 1	1,357,273,764	15.49	88.54	76.54	62.60
	Repeat 2	1,359,512,162	14.15	88.69	75.53	60.03
	Repeat 3	1,345,237,148	13.89	87.76	74.49	59.25
25 °C/Day 3	Repeat 1	1,355,871,585	14.68	88.45	75.57	60.55
	Repeat 2	1,355,865,572	15.32	88.45	76.15	61.80
	Repeat 3	1,369,305,764	16.76	89.33	77.79	64.56
25 °C/Day 7	Repeat 1	1,360,773,065	14.08	88.77	75.43	59.69
	Repeat 2	1,358,078,100	14.47	88.59	75.49	60.33
	Repeat 3	1,360,400,447	14.77	88.74	75.79	60.62
14 °C/Day 7	Repeat 1	1,365,174,801	13.87	89.06	74.83	58.02
	Repeat 2	1,343,423,094	13.14	87.64	73.42	56.65
	Repeat 3	1,356,121,119	13.98	88.47	74.90	58.82
14 °C/Day 10	Repeat 1	1,359,048,106	14.32	88.66	75.42	59.82
	Repeat 2	1,356,076,736	13.49	88.46	74.15	57.69
	Repeat 3	1,363,436,034	13.82	88.94	74.77	58.26

Note: numCovg1, numCovg2, and numCovg3 denote the percentages of cytosine sites covered at depths ≥ 1×, ≥5×, and ≥10×, respectively.

**Table 5:** Methylation status of C sites.

Samples		CG (Mb)	CHG (Mb)	CHH (Mb)	Mean C (%)	Mean CG (%)	Mean CHG (%)	Mean CHH (%)
30 °C Day 3	Repeat 1	468.0	471.0	2643.9	24.80	83.85	66.28	6.95
	Repeat 2	440.4	441.3	2427.2	25.09	82.73	66.16	7.16
	Repeat 3	491.2	493.2	2783.4	24.99	83.19	66.01	7.46
30 °C Day 7	Repeat 1	488.6	490.2	2763.4	25.07	84.28	66.26	7.29
	Repeat 2	447.5	447.4	2521.7	24.84	84.1	66.08	7.01
	Repeat 3	432.1	434.6	2378.4	25.54	84.63	66.77	7.27
25 °C Day 3	Repeat 1	463.7	463.0	2623.9	24.95	83.60	66.56	7.24
	Repeat 2	477.6	475.8	2708.8	24.71	82.80	65.79	7.25
	Repeat 3	528.8	528.7	3007.0	24.59	82.95	65.66	7.11
25 °C Day 7	Repeat 1	451.2	451.9	2561.0	25.02	83.93	66.28	7.36
	Repeat 2	460.2	458.7	2627.4	25.01	83.81	66.24	7.51
	Repeat 3	467.6	468.3	2677.0	25.00	83.87	66.53	7.45
14 °C Day 7	Repeat 1	451.1	450.4	2578.6	23.55	80.21	63.67	6.63
	Repeat 2	411.2	411.7	2334.5	24.18	81.86	64.87	6.85
	Repeat 3	447.1	447.5	2539.1	24.00	81.62	64.70	6.68
14 °C Day 10	Repeat 1	451.2	450.0	2572.0	24.06	82.23	65.12	6.66
	Repeat 2	432.7	433.7	2449.0	24.40	82.44	65.49	6.87
	Repeat 3	445.2	443.3	2551.8	23.92	82.05	64.67	6.7

## Data Availability

Raw RNA-sequencing reads and DNA bisulfite-conversion libraries in this study have been submitted to the National Center for Biotechnology Information and can be accessed via the accession code PRJNA766256.

## Supplementary Materials

The following supporting information can be downloaded at: https://www.mdpi.com/article/10.3390/epigenomes10040067/s1, Figure S1: Water uptake profiles of *M. micrantha* seeds at 14 °C, 25 °C, and 30 °C.
