# Supplementary material for: Cold Stress-Induced DNA Methylation Profiling and Its Association with Terpenoid Biosynthesis in Germinating Mikania micrantha Seeds
Source: Epigenomes. 2026 Oct 12;10(4):67. doi: 10.3390/epigenomes10040067 (PMC13692330; doi:10.3390/epigenomes10040067)
Supplement: Supplementary file 1 [file epigenomes-10-00067-s001.zip › epigenomes-4548329-supplementary.pdf]

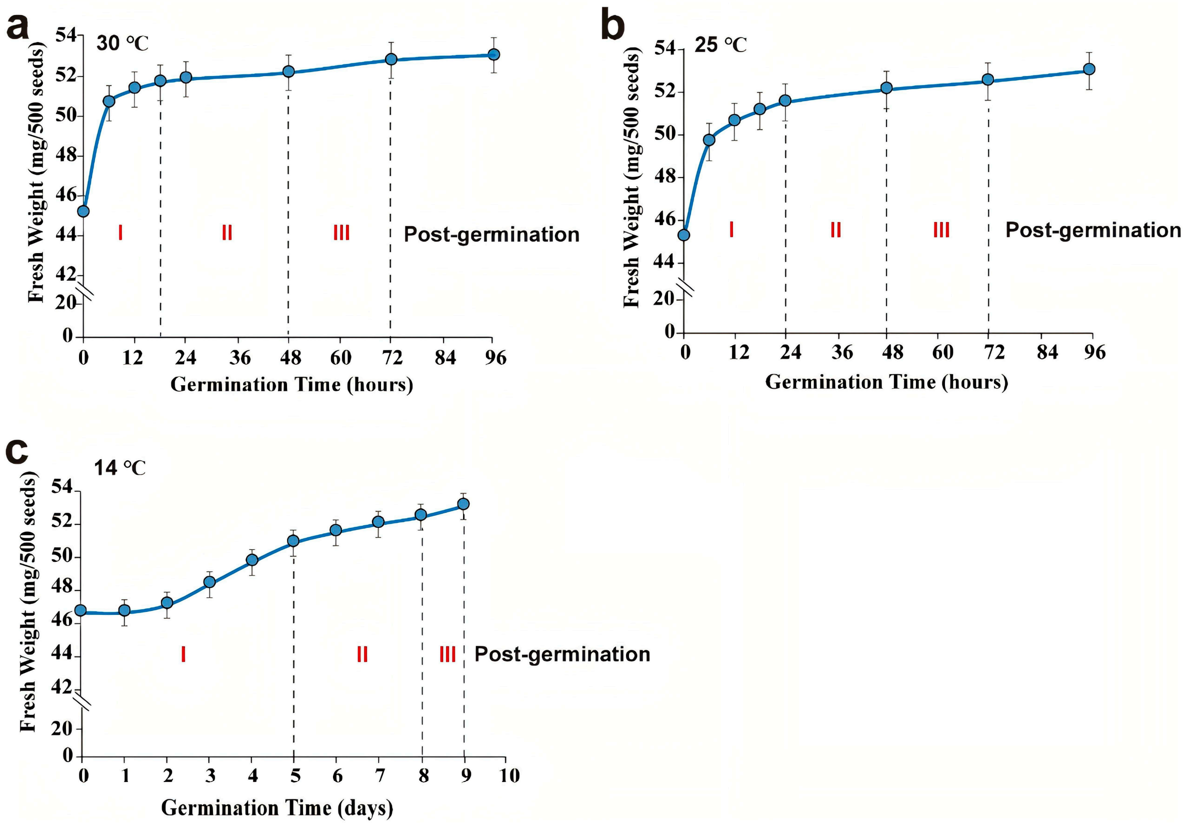

Figure S1. Water uptake profiles of *M. micrantha* seeds at 14 °C, 25 °C, and 30 °C.
